# Advances in the management of pediatric low-grade glioma^☆^

**DOI:** 10.1016/j.neurot.2026.e00952

**Published:** 2026-06-24

**Authors:** Meziane Brizini, Sébastien Perreault, Julie Bennett, Hadeel Hassan

**Affiliations:** a Department of Pediatric Hematology/Oncology/BMT, CancerCare Manitoba, Winnipeg, Canada; b Department of Neurosciences, Division of Child Neurology, University of Montreal, CHU Sainte-Justine, Montreal, Canada; c Division of Hematology/Oncology, The Hospital for Sick Children, Toronto, ON, Canada; d Division of Medical Oncology and Hematology, Princess Margaret Cancer Centre, Toronto, ON, Canada; e Arthur and Sonia Labbatt Brain Tumour Research Centre, The Hospital for Sick Children, Toronto, ON, Canada; f Paul Albrechtsen Institute, CancerCare Manitoba 675 McDermot Avenue, Winnipeg, Manitoba, R3E 0V9, Canada; g Department of Pediatrics and Child Health, University of Manitoba, Winnipeg, Canada

**Keywords:** Low-grade glioma, Targeted therapy, Precision medicine, BRAF inhibitor, MEK inhibitor, Artificial intelligence

## Abstract

Pediatric low-grade gliomas (pLGGs), the most common CNS tumors in children, are increasingly recognized as chronic diseases with prolonged courses and cumulative morbidity. Long-term survival is excellent. Management depends on tumor location and often requires repeated therapy, with risk of long-term functional and neurocognitive impairment. This review synthesizes recent advances in pLGG management, integrating molecular classification, systemic therapies, and emerging diagnostic and surveillance technologies. Treatment advances have been driven by improved molecular characterization, particularly the recognition that most tumors are driven by RAS/MAPK pathway alterations. Integrated histologic-molecular classification has enabled biologically driven risk stratification and adoption of targeted therapies (BRAF, MEK, RAF inhibitors), reshaping treatment for molecularly selected patients. Additionally, recognition of cancer predisposition syndromes and incorporation of germline testing have expanded the scope of clinical decision-making. Emerging technologies including cerebrospinal fluid based liquid biopsy testing, artificial intelligence enabled imaging, advanced metabolic and intraoperative imaging, and digital functional monitoring offer new opportunities to refine diagnosis, surveillance, and response assessments. Despite major progress, challenges remain, including uncertainty about optimal treatment duration and discontinuation, and the management of long-term toxicities from prolonged pathway inhibition. Global disparities in access to molecular diagnostics and targeted therapies, along with financial toxicity from prolonged treatment, hinder equitable precision care. Contemporary pLGG management requires integrating molecular biology, functional outcomes, and long-term surveillance within a multidisciplinary framework. Ongoing research on sequencing, survivorship, and access is essential to improve long-term outcomes.

## Introduction

Pediatric low-grade gliomas (pLGGs) constitute the most common central nervous system (CNS) neoplasm in childhood, accounting for approximately one-third of all pediatric CNS tumors [1]. In the 2021 World Health Organization (WHO) Classification of Tumors of the Central Nervous System, these tumors are broadly grouped into pediatric-type diffuse low-grade gliomas, circumscribed astrocytic gliomas (including pilocytic astrocytoma), and glioneuronal and neuronal tumors. Historically, management was guided by their indolent clinical course, with long-term survivorship data reporting overall survival exceeding 90% at 20 years following diagnosis [1,2]. However, this statistic obscures substantial clinical morbidity related to tumor location, resectability, and biological behavior, with up to half of patients ultimately requiring adjuvant therapy for disease progression [2].

Contemporary practice increasingly views pLGG as a chronic disease, characterized by prolonged disease trajectories, recurrent progression, and repeated therapeutic interventions. This course is associated with cumulative tumor- and treatment-related morbidity that varies substantially by tumor location. Visual impairment affects up to 80% of patients with optic pathway or hypothalamic tumors [2], and endocrine dysfunction occurs in up to 40% [2]. Neurocognitive impairment, most commonly attention deficits reported in 34–55% of survivors [2]. Motor deficits may also be present at diagnosis, though incidence data remain limited [3].

Carboplatin and vincristine regimens or vinblastine monotherapy are the current standard of care in many centers and are commonly used to stabilize disease, with 5-year progression-free survival (PFS) ranging from 40 to 50% for both regimen [[4], [5], [6]]. While effective for disease control in some cases, these approaches imposed significant treatment burden and toxicity without eliminating the need for subsequent salvage therapy in many cases [6,7]. Radiotherapy, once central to salvage management, has been progressively de-emphasized to mitigate late neurocognitive and vascular sequelae. Radiotherapy is generally reserved for refractory disease and is considered only after failure of medical and surgical management, or in carefully selected cases where other therapeutic options are not feasible [8].

The molecular characterization of pLGG was defined by the discovery that most tumors harbor RAS/mitogen-activated protein kinase (MAPK) pathway alterations, most commonly *KIAA1549::BRAF* resulting from tandem duplication at chromosome 7q34 [9]. This established constitutive MAPK activation as the central oncogenic driver and provided a biological framework for the spectrum of alterations subsequently identified, including BRAF p.V600E mutations, *NF1* mutations, *FGFR1/2/3* alterations, and *NTRK* fusions [10]. These discoveries catalyzed a shift toward integrated histological-molecular diagnosis and biomarker-informed treatment, incorporated into the 2021 WHO classification [11].

Advances in management is therefore defined by a recalibration of therapeutic objectives, shifting from survival alone to durable disease control with maximal preservation of function through precision medicine [4,12]. Recent regulatory approvals including combined BRAF and MEK inhibition for BRAF V600E–mutant pLGG and a RAF inhibitor for relapsed BRAF-altered pLGG represent a pivotal inflection point in care highlights the importance of comprehensive tumor molecular profiling to identify actionable alterations [[13], [14], [15]].

Precision medicine introduces new complexities, including uncertainty regarding optimal treatment duration, mechanisms of resistance, rebound growth following discontinuation, and the long-term developmental effects of prolonged pathway inhibition [[16], [17], [18], [19]]. These challenges are further compounded by global disparities in access to advanced diagnostics and targeted therapies [19].

This review provides an expert review and synthesis of advances in medical therapies for pLGG integrating molecular classification with contemporary decision-making. It further addresses management in cancer predisposition syndromes, emerging diagnostic and surveillance technologies, and health-system challenges shaping current research priorities.

## Molecular landscape

Contemporary management of pLGG is increasingly structured around integrated histological-molecular diagnosis, because histology alone does not reliably predict clinical behavior, therapeutic vulnerability, or relevant risk modifiers [11,12,20]. Across pLGGs, recurrent alterations typically converge on activation of the MAPK pathway. However, the mechanism of activation—whether fusion-driven dimeric signaling, activating single-nucleotide variants (SNVs), copy-number alterations, or syndromic predisposition—has practical implications for diagnostic work-up, prognostication, and systemic therapy selection. Therefore, comprehensive molecular profiling using platforms capable of detecting SNVs, copy-number changes, and structural variants (including gene fusions and internal tandem duplications [ITDs]) is integral to contemporary practice (Table 1) [11]. Most pLGGs are characterized by a single dominant driver alteration; however, additional alterations in tumor suppressor genes (e.g., TP53, ATRX, CDKN2A/B) may signify more aggressive tumor biology [[20], [21], [22], [23], [24]].

**Table 1:** Recurrent molecular alterations in pLGG and their clinical implications.

Molecular class	Representative alterations	Mechanism of MAPK activation	Common histologies	Typical locations	Prognostic features	Therapeutic relevance	Molecular class
BRAF fusion–driven	*KIAA1549::BRAF*	Constitutive dimer-dependent RAF signaling	Pilocytic astrocytoma	Posterior fossa, optic pathway	Indolent course, prolonged survival	MEK inhibition; resistance to type I BRAF inhibitors	Requires fusion-sensitive assays
BRAF V600E–driven	BRAF V600E ± CDKN2A loss	Constitutive monomeric RAF activation	PXA, ganglioglioma, diffuse LGG	Supratentorial	Higher progression risk; adverse with CDKN2A loss	BRAF + MEK inhibition	IHC reliable; assess CDKN2A status
NRTK-driven	FGFR1/2/3 SNVs, ITDs, fusions; NTRK fusions	Upstream receptor tyrosine kinase activation	DNET, RGNT, selected PA, diffuse LGG	Variable; midline for FGFR1 point mutations; cerebral for FGFR1-ITD	Variable; modified by co-alterations	Emerging FGFR/TRK inhibitors	RNA-based testing often required
NF1-driven	NF1 loss	Loss of RAS-GAP activity	NF1-associated LGG, optic pathway glioma	Optic pathway, brainstem	Typically indolent; context-dependent	MEK inhibition	Germline testing important
MYB/MYBL1-driven	MYB/MYBL1 fusions/truncations	Distinct transcriptional programs (non-MAPK)	Diffuse astrocytoma MYB/MYBL1-altered, angiocentric glioma	Cerebral hemispheres (frontal, temporal, parietal); rare brainstem	Excellent prognosis; rare malignant transformation	Not MAPK-targetable	Truncations may be missed by fusion-only assays
IDH-mutant	IDH1/2 mutations ± ATRX/TP53 loss or 1p/19q codeletion	Neomorphic IDH activity leading to altered cellular metabolism and epigenetic dysregulation (non-MAPK)	Astrocytoma IDH-mutant; oligodendroglioma IDH-mutant 1p/19q-codeleted	Predominantly supratentorial, hemispheric	Variable; often indolent initially with risk of late progression; oligodendroglioma generally favorable; CDKN2A/B deletion defines grade 4 behavior	Classified as adult-type diffuse gliomas in WHO 2021; limited pediatric-specific trial data; IDH inhibitors under investigation	IDH1 R132H IHC appropriate in adolescents; sequencing required if IHC negative; rare in young children

**Abbreviations:***BRAF*, v-raf murine sarcoma viral oncogene homolog B; *CDKN2A*, cyclin-dependent kinase inhibitor 2A; *DNET*, dysembryoplastic neuroepithelial tumor; *FGFR*, fibroblast growth factor receptor; *IHC*, immunohistochemistry; *IDH*, isocitrate dehydrogenase; *ITD*, internal tandem duplication; *LGG*, low-grade glioma; *MAPK*, mitogen-activated protein kinase; *MEK*, mitogen-activated protein kinase kinase; *NF1*, neurofibromatosis type 1; *NTRK*, neurotrophic tyrosine receptor kinase; *PA*, pilocytic astrocytoma; *PXA*, pleomorphic xanthoastrocytoma; *RAF*, rapidly accelerated fibrosarcoma; *RGNT*, rosette-forming glioneuronal tumor; *SNV*, single-nucleotide variant; *TRK*, tropomyosin receptor kinase.

### BRAF alterations

BRAF alterations are the most common oncogenic events in pLGG and occur predominantly as gene fusions, or less frequently as activating point mutations. BRAF fusions, most commonly *KIAA1549::BRAF*, arise through tandem duplication at chromosome 7q34 and account for 60–80% of pilocytic astrocytomas (PAs) and approximately 15% of other pLGGs [11]. These tumors typically arise in the posterior fossa, followed by supratentorial locations including the optic pathway [11,25]. BRAF fusion-positive tumors demonstrate constitutive, RAS-independent, dimeric RAF signaling, resulting in sustained MAPK pathway activation. Clinically, these tumors are often indolent with favorable outcomes, although prognosis remains strongly influenced by tumor location and resectability [25,26].

Accurate detection requires fusion-sensitive assays, including Ribonucleic Acid (RNA) based sequencing or targeted PCR approaches for common breakpoints. This is because conventional hotspot deoxyribonucleic acid (DNA) panels may miss fusions and fluorescence in situ hybridization (FISH) may be unreliable depending on probe design and breakpoint location. Importantly, the dimer-dependent signaling conferred by BRAF fusions renders these tumors intrinsically resistant to first-generation type I BRAF inhibitors (such as dabrafenib and vemurafenib) through paradoxical MAPK pathway activation, a distinction with direct therapeutic implications [4].

In contrast, activating BRAF point mutations, most commonly BRAF p.V600E (15–20% of pLGG), result in constitutive monomeric kinase activation and define a biologically and clinically distinct subgroup [4,20,22,25]. BRAF p.V600E pLGGs are more often supratentorial and are frequently observed in pleomorphic xanthoastrocytoma (60–80%) and ganglioglioma (20–30%), and less commonly in pilocytic astrocytoma (∼5%) [11]. Compared with BRAF fusion–positive tumors, BRAF p.V600E–mutant lesions are associated with inferior progression-free survival (10-year PFS 27% vs 60% for wild-type) and poorer response to conventional chemotherapy [27]. Prognosis is further modified by co-occurring genetic alterations, most notably homozygous deletion of CDKN2A/B, which confers increased risk of aggressive behavior and is an independent adverse prognostic factor [20,25,28].

### FGFR alterations

Alterations involving fibroblast growth factor receptors (FGFRs) represent a heterogeneous but clinically relevant class of MAPK-activating events in pLGG. These include activating SNVs, ITDs, and gene fusions, all of which can result in ligand-independent receptor activation and downstream MAPK signaling [[20], [21], [22], [23], [24]]. FGFR alterations are observed across a range of pediatric CNS tumors and contribute to the molecular diversity of pLGG. Although FGFR-altered tumors are generally indolent, clinical behavior is context-dependent and influenced by co-occurring genetic events.

Comprehensive profiling capable of detecting the full spectrum of FGFR alterations is required for accurate classification, as standard DNA panels may inadequately capture ITDs or fusions; RNA sequencing is particularly important for fusion detection [21]. Emerging evidence also suggests that FGFR1 hotspot mutations (particularly N546K and K656E) are associated with spontaneous intra-tumoral hemorrhage in pediatric and young adult LGG, especially in diencephalic locations, although the mechanism remains unclear and the association appears specific to FGFR1 point mutations rather than all FGFR alteration types [29]. FGFR single-nucleotide variants (SNVs) may co-occur with other RAS/MAPK pathway alterations but are independently sufficient to drive gliomagenesis. However, the clinical and prognostic implications of these co-occurring events remain unclear [21].

### NTRK alterations

Neurotrophic tyrosine receptor kinase (NTRK) gene fusions define a rare but therapeutically actionable subset of pLGG. These alterations result in constitutive tropomyosin receptor kinase (TRK) activation with downstream MAPK signaling and are enriched in infantile gliomas and selected rare histologic subtypes. NTRK fusions, are identified in approximately 2% of pediatric gliomas overall [5]. The distribution of NTRK family members varies by age: NTRK2 fusions are more common in pediatric cases (including cerebral diffuse LGGs), whereas NTRK1 fusions are more frequent in adults and have been described in desmoplastic infantile ganglioglioma; NTRK3 fusions occur less commonly across the pediatric age spectrum [30].

NTRK fusion–positive pLGG also demonstrates age-dependent heterogeneity. Infantile tumors often harbor the fusion as an isolated driver, whereas pediatric and adult cases may show additional alterations (e.g., ATRX, PTEN, CDKN2A/B, TP53, TERT promoter) that influence tumor grade and clinical behavior [30]. Accurate detection requires RNA-based fusion assays or comprehensive sequencing platforms with validated fusion capture, given the diversity of fusion partners [4].

### Other molecular alterations

Beyond MAPK-pathway drivers, additional alterations contribute to molecular heterogeneity and define diagnostically important entities. Activating RAS mutations (including KRAS and less commonly HRAS) are reported in selected pLGG contexts; KRAS mutations have been described in tectal gliomas, although these tumors are infrequently biopsied and the full molecular spectrum remains incompletely defined [31,32].

Rearrangements involving MYB or MYBL1 define WHO-recognized entities, including diffuse astrocytoma, MYB- or MYBL1-altered and angiocentric glioma [33]. These alterations involve structural events leading to deregulated MYB family transcriptional activity and overexpression of MYB(L1) [34]. Notably, truncations without productive in-frame fusions can also be oncogenic, with implications for diagnostic strategies that rely solely on fusion detection [34]. MYB/MYBL1-altered tumors are typically indolent with excellent prognosis, although rare malignant transformation has been reported in association with additional alterations such as TERT or TP53 mutations [35].

A subset of pediatric gliomas harbor IDH1 or IDH2 mutations with strong age dependence; approximately 0.5% in children aged 0–9 years, rising to up to 16% in adolescents aged 10–21 years [36]. These tumors are classified as adult-type diffuse gliomas in WHO 2021, and National Comprehensive Cancer Network (NCCN) Pediatric CNS guidance directs management under adult CNS cancer frameworks [36,37]. IDH-mutant gliomas show co-alterations typical of adult disease (ATRX/TP53 in astrocytoma; 1p/19q codeletion in oligodendroglioma). Although uncommon, reported frequencies vary (5–16% across some pLGGs) [38]. Collectively, these tumors represent an important group within the pLGG spectrum and highlight the need for age-appropriate molecular interpretation within integrated diagnostic frameworks.

## Systemic therapy in the molecular era

The delineation of recurrent molecular drivers in pLGG has enabled a transition from empiric cytotoxic therapy based on histology alone, toward pathway-directed treatment and molecularly informed risk stratification [20]. Targeted therapies now occupy a central role in management for selected patient populations. The major molecularly targeted therapies and practical considerations are summarized in Table 2, and ongoing targeted therapy trials are summarized in Table 3.

**Table 2:** Molecularly Targeted Therapies for pLGGs: Regulatory Status and Key Considerations.

Molecular Target	Drug	Regulatory Status	Key Considerations
BRAF V600E	Dabrafenib + trametinib	Approved (FDA/EMA/Canada)	First-line pLGG; superior to chemotherapy (ORR 47% vs 11%); AEs: Pyrexia (68%), headache, rash, weight gain; optimal duration unknown
BRAF fusion	Trametinib	Not pLGG-specific (FDA/EMA/Canada)	Second-line option when treatment required; avoids paradoxical MAPK activation; not yet approved as a first line AEs: Acneiform rash, GI symptoms, elevated CPK; rebound risk on discontinuation
BRAF fusion or V600E	Tovorafenib	FDA: Accelerated approval (April 2024); EMA/Canada: Under review	Active in fusion and V600E tumors; CNS-penetrant; ORR 51% AEs: Rash (93%), hair color changes, fatigue, hemorrhage, reduced growth velocity; frontline trials ongoing
NF1-associated pLGG	Selumetinib	FDA/EMA/Canada: Approved for NF1 PN only (off-label for pLGG)	Could be used off-label for NF1-associated pLGG but access remains difficult off study. AEs: GI symptoms, elevated CPK, acneiform rash, paronychia; cardiac and ocular monitoring required
NTRK fusion	Larotrectinib	Approved (FDA/EMA/Canada) – tumor-agnostic	Rapid and durable responses in pLGG; AEs: Elevated transaminases, anemia, fatigue; generally well tolerated; resistance may develop
NTRK fusion	Entrectinib	FDA: Tumor-agnostic (>1 month, Oct 2023); EMA: ≥12 years; Canada: Approved	Rapid and durable responses; AEs: Weight gain (35%), pathological fractures, anemia, dizziness/ataxia, neurocognitive effects
NTRK fusion (post-TRK)	Repotrectinib	FDA: Accelerated approval ≥12 years (June 2024); EMA/Canada: Under review	Option after TRK inhibitor resistance; ORR 50% in TKI-pretreated; limited pediatric pLGG data
FGFR alteration	Erdafitinib	Not pLGG-specific (FDA/EMA/Canada)	Investigational; modest activity; AEs: SCFE and accelerated linear growth are major pediatric toxicities; hyperphosphatemia
mTOR pathway (TSC-SEGA)	Everolimus	Approved (FDA/EMA/Canada) for TSC-associated SEGA	Standard of care for TSC-associated SEGA specifically; AEs: Stomatitis, infections, hyperlipidemia; requires continuous treatment
IDH1/2 mutation	Vorasidenib	FDA: Approved ≥12 years (Aug 2024); EMA/Canada: Under review	Post-resection or residual disease; pediatric data limited; AEs: Elevated transaminases, fatigue; benefit after GTR unknown

**Abbreviations***AEs*, adverse events; *BRAF*, B-Raf proto-oncogene; *CNS*, central nervous system; *CPK*, creatine phosphokinase; *EMA*, European Medicines Agency; *FDA*, US Food and Drug Administration; *FGFR*, fibroblast growth factor receptor; *GI*, gastrointestinal; *GTR*, gross total resection; *IDH*, isocitrate dehydrogenase; *KRAS*, Kirsten rat sarcoma viral oncogene homolog; *LGG*, low-grade glioma; *MAPK*, mitogen-activated protein kinase; *MEK*, mitogen-activated protein kinase.

**Table 3:** Ongoing targeted therapy clinical trials.

NCT ID	Phase	Population	Primary Endpoint	Treatment/*Drug Class*	Age (years)	Recruitment Status
NCT06666348	1/2	Untreated MAPK-activated pLGG	- Determine MTD of Mirdametinib + vinblastine. - Determine ORR of Mirdametinib + vinblastine.	- Mirdametinib + vinblastine - *MEKi + Chemo*	2–25	Active/Recruiting
NCT06381570	Pilot	R/R pLGG	- Determine MTD/RP2D of vinblastine + tovorafenib.	- Vinblastine + tovorafenib - *RAFi + Chemo*	≤25	Active/Recruiting
NCT06712875	1/2	R/R MAPK-activated gliomas (LGG & HGG)	- Determine MTD of trametinib + nivolumab.	- Trametinib + nivolumab - *MEKi + ICI*	1–26	Active/Recruiting
NCT07004075	3	Untreated or R/R pLGG & adult LGG	- Compare the PFS of Luvometinib versus chemotherapy.	- Luvometinib - *MEKi*	2–18	Active/Not yet recruiting
NCT05566795	3	Untreated pLGG	- Determine the ORR of tovorafenib vs SoC chemotherapy.	- Tovorafenib vs chemo (SoC) - *RAFi vs Chemo*	≤25	Active/Not recruiting
NCT04485559	1	R/R gliomas (LGG & HGG)	- Determine MTD/RP2D of trametinib + everolimus.	- Trametinib + everolimus - *MEKi + mTORi*	1–25	Suspended^a^
NCT01089101	1/2	R/R pLGG	- Determine MTD/RP2D of selumetinib. - Determine ORR sustained for 8 weeks. - Determine disease stabilization rates.	- Selumetinib monotherapy - *MEKi*	3–21	Active/Not recruiting
NCT06521567	1/2	Pediatric and young adult newly or R/R tumors (LGG included among other solid cancer)	- Determine MTD/RP2D of cobolimab + Dostarlimab. - Determine the treatment adverse events of cobolimab + Dostarlimab.	- Cobolimab + Dostarlimab - *TIM-3i + ICI*	0–21	Active/Not recruiting
NCT04775485	2	R/R pLGG	- Determine ORR of tovorafenib monotherapy. - Determine the adverse event rate with tovorafenib.	- Tovorafenib monotherapy - *RAFi*	0.5–25	Active/Recruiting but not for pLGG
NCT03871257	3	Untreated NF1-associated LGG	- Determine PFS for selumetinib vs carboplatin + vincristine. - Determine the visual acuity improvement rate.	- Selumetinib vs carboplatin + vincristine - *MEKi v. Chemo*	2–21	Active/Not recruiting
NCT04166409	3	Untreated non-NF1/non-BRAF V600E LGG (no chemotherapy/Radiation)	- Determine EFS for selumetinib vs carboplatin + vincristine.	- Selumetinib vs carboplatin + vincristine - *MEKi v. Chemo*	2–21	Active/Recruiting
NCT04576117	3	Non-NF1, non-BRAFV600E, non-TSC, R/R pLGG	- Determine MTD/RP2D of selumetinib and vinblastine combination. - Determine the EFS of selumetinib and vinblastine combination.	- Selumetinib + vinblastine vs selumetinib alone - *MEKi v. Chemo*	2–25	Active/Not recruiting
NCT02358187	2	R/R unresectable HLA-A2+ LGG	- Determine the tumor shrinkage rate and stable disease rate of GAA/TT peptide vaccine and poly-ICLC.	- GAA/TT peptide vaccine and poly-ICLC - *Vaccine*	1–22	Active/Recruiting

**Abbreviations:** EFS, Event-free survival; *GAA*, glioma-associated antigens; *HGG*, high-grade glioma; *HLA*, human leukocyte antigen (HLA-A2+, HLA-A^a^02 positive); *ICI*, immune checkpoint inhibitor; *LGG*, low-grade glioma; *MAPK*, mitogen-activated protein kinase; *MEKi*, mitogen-activated protein kinase kinase inhibitor; *mTORi*, mammalian target of rapamycin inhibitor; MTD, Maximum tolerated dose; *NF1*, neurofibromatosis type 1; ORR, Objective response rate; PFS, Progression-free survival; *pLGG*, pediatric low-grade glioma; *poly-ICLC*, polyinosinic–polycytidylic acid stabilized with poly-l-lysine and carboxymethylcellulose; *RAFi*, RAF kinase inhibitor; RP2D, Recommended phase 2 dose; *R/R*, recurrent or refractory; *SoC*, standard of care; *TIM-3*, T-cell immunoglobulin and mucin domain-containing protein 3; *TSC*, tuberous sclerosis complex; *TT*, tetanus toxoid.

a Pending protocol amendment to transition to commercial drug supply.

### RAS/MAPK pathway

MEK inhibitors have demonstrated activity across MAPK-driven pLGGs, including Neurofibromatosis type 1 (NF1) associated tumors. Selumetinib achieved objective response rates of 36–40% in recurrent or progressive pLGG [4]. This supported the initiation of a phase 3 trials (NCT04166409, NCT03871257) comparing selumetinib with standard chemotherapy in upfront settings that unfortunately closed early because of poor accrual [39]. Trametinib monotherapy has also demonstrated durable disease control in refractory disease, although treatment discontinuation due to toxicity remains a clinically relevant limitation [40]. Additional MEK inhibitors are also under investigation (Table 3).

For BRAF V600E–mutant pLGG, combined BRAF and MEK inhibition with dabrafenib and trametinib has emerged as a practice-defining approach. A phase II study demonstrated superior response rates and progression-free survival compared with vincristine–carboplatin chemotherapy with an ORR of 47% versus 11% (p < 0.001) and a median PFS of 20.1 months versus 7.4 months (p < 0.001), respectively. This led to FDA approval as first-line therapy in March 2023 [14].

First-generation type I BRAF inhibitors are contraindicated in BRAF fusion–driven tumors due to paradoxical MAPK activation [25]. Tovorafenib, a CNS-penetrant type II RAF inhibitor, overcomes this limitation and demonstrated substantial activity in the FIREFLY-1 trial (overall response rate (ORR) of 53% and a median PFS of 13.9 months), leading to FDA accelerated approval in April 2024 for relapsed or refractory RAF-altered pLGG [41]. Ongoing trials (LOGGIC/FIREFLY-2) are evaluating tovorafenib as a frontline alternative to chemotherapy [42].

The optimal duration of MAPK-directed therapy remains undefined. Tumor rebound following treatment discontinuation is well described and is thought to reflect rapid MAPK reactivation [16]. A subset of patients, however, remains progression-free after cessation, suggesting durable disease control may be achievable in selected cases [18,23]. Canadian consensus recommendations propose treatment durations of approximately 36 months for pLGG with BRAF p.V600E, with gradual tapering to mitigate rebound risk; in general, MEK inhibitors are tapered first, followed by BRAF inhibitors, with close radiologic surveillance during dose reduction [18]. Emerging data in 2026 from ongoing analyses comparing MEK inhibitor tapering versus abrupt discontinuation are expected to further inform optimal treatment duration and de-escalation strategies.

The oncogene-induced senescence (OIS), a tumor-suppressive mechanism that limits uncontrolled cellular proliferation, is paradoxally induced by MAPK pathway activation in pLGGs [43,44]. OIS is mediated through activation of cell-cycle regulatory pathways, including p16INK4a and p53 signaling, and may explain the long-term stability and indolent behavior [43]. The impact of prolonged MAPK pathway inhibition on oncogene-induced senescence (OIS) in pLGG remains unknown and it represents an important gap in understanding what could be the long-term biological consequences of targeted therapies.

Toxicities require proactive management. Pyrexia is common with dabrafenib–trametinib combination therapy, while cutaneous toxicities occur frequently with MEK and RAF inhibitors. Tovorafenib is associated with frequent but generally low-grade rash and growth suppression with recovery after treatment interruption for most patients [14]. Long-term effects on growth and cardiovascular health remain under investigation [41,42].

### FGFR alterations

FGFR alterations represent a heterogeneous subset of pLGG with emerging therapeutic relevance. Early-phase studies suggest activity of FGFR inhibitors in FGFR-altered pediatric gliomas, although response rates are modest and pediatric-specific data remain limited [9,21]. Erdafitinib has demonstrated disease control in a subset of patients in Pediatric MATCH, whereas data for pemigatinib remain sparse [[45], [46], [47]]. FGFR inhibitors are associated with significant toxicity that constrain their use mainly cutaneous and nail toxicities and hyperphosphatemia [47,48]. In children, the on-target skeletal toxicities, including slipped capital femoral epiphysis and abnormal linear growth, may require careful orthopedic monitoring [25,49]. At present, FGFR inhibitors remain investigational in pLGG and are not standard of care.

### NTRK alterations

TRK inhibitors have demonstrated robust activity in NTRK fusion–positive pediatric CNS tumors. Recent trials demonstrated that larotrectinib (Phase I/II SCOUT trials, Phase II NAVIGATE trial) and entrectinib (Phase I/II STARTK-NG trial) have achieved reasonable durable disease control in children and young adults CNS tumors, supporting regulatory approvals across pediatric age groups. Respectively, they achieved an ORR of 46% and 54% and a disease control rate (DCR) of 92% and 81.3% [50,51]. Repotrectinib has received accelerated approval for resistant disease and remains under active investigation [52]. Given the efficacy of TRK inhibition, identification of NTRK fusions is clinically critical despite rarity [53,54].

### Other molecular alterations

For less common molecular alterations encountered in pLGG, management remains primarily driven by surgical and clinical considerations, with limited roles for targeted systemic therapy. Activating RAS mutations (KRAS, HRAS, NRAS) occur in a subset of pLGGs and have been associated with worse outcomes compared with rearrangement-driven tumors; however, no direct RAS-targeted therapies are currently approved [55]. In the absence of a defined actionable target, systemic treatment typically relies on downstream MAPK pathway inhibition with MEK inhibitors (e.g., selumetinib, trametinib), while maximal safe resection remains the cornerstone of management when anatomically feasible [12].

Tumors harboring MYB or MYBL1 alterations typically exhibit indolent clinical behavior with excellent outcomes following surgical resection [56]. No targeted therapies are currently available, and gross total resection is often curative. In unresectable or progressive cases, management is individualized according to symptom burden and tumor trajectory. Although prognosis is generally favorable, co-occurring alterations such as CDKN2A/B deletion may increase progression risk, and rare malignant transformation has been reported [57].

For IDH-mutant gliomas, vorasidenib, a brain-penetrant dual IDH1/2 inhibitor, received FDA approval in August 2024 for adults and pediatric patients ≥12 years with grade 2 IDH-mutant astrocytoma or oligodendroglioma with residual or recurrent disease following surgery [58]. In the INDIGO trial, vorasidenib significantly improved progression-free survival (median 27.7 vs 11.1 months; HR 0.39, P < 0.001), delayed time to next intervention (HR 0.26, P < 0.001), reduced tumor growth rate, and improved seizure control without adverse effects on quality of life or neurocognition [59]. Patients without residual disease after gross total resection were excluded; therefore, benefit in this subgroup remains unknown [59]. Pediatric-specific efficacy data are limited because IDH mutations are rare in younger children and the trial population only included one pediatric patient, who was randomized to a placebo. Until pediatric-specific data mature, surgical management and adult-informed treatment frameworks remain central to care for this small subset of pediatric patients with IDH-mutant gliomas [36]. Ongoing studies are expected to further inform management in the near future.

## Cancer predisposition syndromes

Approximately 11% of pediatric gliomas harbor germline pathogenic variants identified through paired tumor–normal sequencing [60,61]. Recognition of cancer predisposition influences treatment selection, toxicity thresholds, and surveillance strategies. Germline testing is recommended for specific tumor–syndrome associations and in the presence of high-risk clinical or molecular features [[60], [61], [62]].

### Detection of cancer predisposition in pediatric low-grade glioma

Beyond low grade glioma (LGG) subtypes with established associations to cancer predisposition syndromes, broader germline evaluation should be considered in pLGG presenting with high-risk clinical or molecular features. Indicators prompting germline testing include diagnosis before two years of age, multifocal disease, a personal or family history of cancer, the presence of café-au-lait macules or other syndromic features, and tumors with atypical molecular profiles such as hypermutation or biallelic tumor suppressor inactivation [60,61].

Importantly, reliance on personal and family history alone fails to identify a substantial proportion of pathogenic germline variants, as many children with inherited cancer predisposition lack overt clinical features or known familial cancer history at diagnosis [61,62]. Tumor-only sequencing may raise suspicion for an underlying germline alteration but is insufficient to distinguish somatic from inherited variants; confirmatory germline testing using blood or saliva is therefore essential. Tumor-only approaches are estimated to miss approximately 8–10% of pathogenic germline variants [60,61].

Identification of cancer predisposition has direct and clinically actionable consequences. Germline status informs surveillance strategies for additional malignancies, guides therapeutic decision-making including avoidance of radiotherapy in Li-Fraumeni syndrome and prioritization of MEK inhibition in NF1 and enables cascade testing of at-risk family members. It also identifies patients at increased risk of malignant transformation and treatment-related toxicity.

Ongoing research aims to refine algorithms for identifying which pLGGs warrant germline testing beyond established high-risk features, develop rapid-turnaround germline assays capable of informing upfront treatment decisions, and better define the biological and therapeutic implications of biallelic tumor suppressor inactivation, which is more common in younger children and confers distinct vulnerabilities. Future priorities include broader implementation of paired tumor and germline sequencing, integration of germline status into clinical trial stratification, and development of international registries to optimize surveillance and long-term outcomes in germline-positive patients [[63], [64], [65]].

### Neurofibromatosis type 1

Neurofibromatosis type 1 (NF1) represents the most common cancer predisposition syndrome associated with pLGG, with approximately 15–20% of affected individuals developing LGGs during childhood. Nearly 80% of these tumors involve the optic pathway, with the remainder arising in the brainstem, cerebellum, or other midline locations [23,66]. NF1-associated pLGGs are driven by germline loss of neurofibromin, resulting in constitutive RAS/MAPK pathway activation, and typically exhibit an indolent growth pattern distinct from sporadic gliomas.

Contemporary management emphasizes a conservative, function-first approach. Therapeutic intervention is guided by documented clinical deterioration, most commonly visual decline, rather than radiographic progression alone, as a substantial proportion of optic pathway gliomas remain asymptomatic or demonstrate spontaneous stabilization or regression [67]. Accordingly, surveillance strategies prioritize serial ophthalmologic assessment, with MRI reserved for symptomatic patients or those unable to undergo reliable visual testing. Radiotherapy is contraindicated whenever possible due to increased risks of vasculopathy, including Moyamoya syndrome, and secondary malignancies [67].

MEK inhibition has transformed systemic therapy for progressive NF1-associated pLGG. The use of selumetinib in NF1-associated pLGG is still under investigation but it has demonstrated promising results compared with conventional chemotherapy [68]. Ongoing studies aim to refine predictors of progression, identify biomarkers of visual outcome, and determine whether early intervention can improve long-term functional preservation [69,70].

### Tuberous sclerosis complex

In tuberous sclerosis complex (TSC), LGGs most commonly present as subependymal giant cell astrocytomas (SEGAs), driven by biallelic inactivation of TSC1 or TSC2 and constitutive activation of the mTOR pathway. Management has been fundamentally altered by the advent of mTOR inhibitors, which have largely supplanted surgical resection as first-line therapy except in cases of acute hydrocephalus or mass effect [71].

Clinical trials (including the EXIST-1 trial) and long-term extension studies have demonstrated durable tumor control with everolimus and sirolimus during active treatment, with significant reductions in tumor volume and stabilization of disease over many years [72,73]. However, tumor regrowth following treatment discontinuation is common, necessitating prolonged therapy in many patients. This introduces challenges related to chronic toxicity, including metabolic complications, immunosuppression, and potential effects on growth and development [73].

Current research priorities include optimization of dosing strategies to balance long-term efficacy and toxicity, identification of biomarkers predicting durable response, and development of evidence-based criteria for treatment discontinuation in select patients [74,75]. Notably, late treatment failure has been reported despite prolonged disease control, underscoring the need for ongoing surveillance even in long-term responders [76]. Future priorities include dose-optimization strategies to balance long-term efficacy and toxicity, along with identification of biomarkers that predict progression and durable response. Evidence-based criteria are also needed to support safe treatment de-escalation or discontinuation in selected patients and to evaluate long-term developmental and metabolic outcomes following early-life mTOR inhibition [77].

### Li-Fraumeni syndrome

Li-Fraumeni syndrome (LFS), caused by germline TP53 mutations, poses substantial challenges in the management of pLGG due to the markedly increased risk of malignant transformation and treatment-related secondary malignancies. Low-grade diffuse astrocytomas arising in this context frequently harbor *IDH* mutation often the noncanonical IDH1 R132C variant, represents a distinct molecular subtype compared with sporadic counterparts. These LFS-associated LGGs demonstrate a significantly higher propensity for progression to high-grade disease, necessitating intensified surveillance and individualized, risk-adapted treatment strategies [78,79].

Management should prioritize maximal safe surgical resection. The role of radiotherapy should be considered on an individual, case-by-case basis, with close clinical and radiographic surveillance. Based on molecular profiling, the addition of targeted therapy should be considered when actionable alterations are identified, particularly in cases of IDH-mutant LGG. The updated Toronto Protocol recommends comprehensive surveillance, including annual whole-body MRI and dedicated brain MRI beginning in infancy, enabling early detection of malignancies at potentially curable stages [80]. Prospective data demonstrate that whole-body MRI detects approximately 40.5% of cancers asymptomatically, with 86% identified at localized, potentially curable stages [81].

Future directions in LFS-associated pLGG include refinement of risk stratification to identify tumors at greatest risk of malignant transformation, development of pharmacologic prevention strategies, and integration of emerging technologies such as liquid biopsy for early detection. Although these approaches are not specific to pLGG, they are likely to have broad implications for TP53-driven pediatric brain tumors [[82], [83], [84]].

### Constitutional mismatch repair deficiency

Constitutional mismatch repair deficiency (CMMRD) is a rare germline cancer predisposition syndrome characterized by tumor hypermutation and microsatellite instability [63]. Although uncommon in pLGG (prevalence <1%), CMMRD-associated tumors are associated with poor outcomes following conventional therapy. Immune checkpoint inhibitors (ICIs), including nivolumab and pembrolizumab, have demonstrated significant activity in CMMRD-associated LGG, likely driven by high neoantigen burden. In a landmark multi-cohort study, Negm et al. reported that ICI therapy improved 5-year overall survival in pediatric CMMRD LGG from 10.9% to 44.7% (p < 0.0001) [63]. On multivariable analysis, ICI treatment was the only independent predictor of improved survival (HR 0.4, 95% CI 0.3–0.7; p = 0.0017), irrespective of age or germline status [63]. In contrast, ICIs have limited efficacy in sporadic pLGG without CMMRD or hypermutation, with pooled objective response rates of 4.1% in unselected pediatric CNS tumors. Responses are largely confined to molecularly defined subgroups characterized by hypermutation.

### Noonan syndrome

Noonan syndrome (NS) is an autosomal dominant RASopathy most commonly associated with germline *PTPN11* mutations [85]. NS is associated with an increased risk of childhood cancer, including a predisposition to LGGs and dysembryoplastic neuroepithelial tumors (DNETs) [85,86]. Germline *PTPN11* alterations typically result in missense gain-of-function mutations, leading to activation of SHP2 and prolonged activation of the RAS/MAPK pathway [87]. In a literature review by Lodi et al., 26 of 28 published NS-associated CNS tumors were DNETs or LGGs (92.9%). Among these, DNETs accounted for up to 40%, compared with less than 1% of CNS tumors in the general population [86]. NS-associated LGGs and glioneuronal tumors may be underdiagnosed before cancer genomic profiling, and there are currently no recommendations for routine CNS tumor surveillance in patients with NS [88]. MEK inhibitors, such as trametinib and selumetinib, may be useful in RAS/MAPK-altered NS-associated LGG, while combined dabrafenib and trametinib may be considered when a *BRAF* V600E mutation is identified [89].

## Emerging diagnostic, therapeutic, and monitoring technologies

The management of pLGG is shifting from reactive surveillance toward proactive, precision monitoring, reflecting the increasing importance of molecular characterization and longitudinal response assessment in a chronic disease context. Emerging approaches, including cerebrospinal fluid (CSF) based liquid biopsy, artificial intelligence (AI) enabled imaging, advanced metabolic and intraoperative techniques, and digital functional monitoring, aim to address limitations of conventional diagnostics. Together, these strategies enable less invasive molecular profiling, improve reproducibility of disease assessment, and support integrated evaluation of tumor behavior and functional outcomes. These tools are particularly relevant in pLGG, where tumors often arise in surgically inaccessible regions and treatment response may be cytostatic rather than cytoreductive.

### Liquid biopsy

The application of liquid biopsy in CNS tumors has historically been constrained by limited shedding of circulating tumor DNA (ctDNA) into peripheral blood due to the blood–brain barrier. However, Wang et al. (2015) demonstrated that CSF analysis could serve as an alternative approach, as CSF is enriched with tumor DNA [90]. Escudero et al. (2020) further showed that CSF analysis can support the management, molecular profiling, and monitoring of patients with CNS tumors in a cohort of patients with medulloblastoma [91]. CSF-based liquid biopsy has since progressed from a research tool to an emerging clinical application, with the 2025 NCCN guidelines recommending CSF tumor-derived DNA testing alongside cytology in adult CNS tumors to improve detection of residual disease [[92], [93], [94], [95]]. Although pediatric-specific guidelines are lacking, select centers have begun incorporating CSF ctDNA analysis into clinical workflows for children with CNS tumors [92].

Liquid biopsy is particularly attractive in pLGG, where tumors frequently arise in challenging surgically inaccessible regions and molecular confirmation is increasingly required to guide targeted therapy [96]. However, pLGG present distinct technical challenges compared with high-grade gliomas, including lower overall ctDNA detection rates (approximately 40–50%) and difficulty detecting gene fusions, particularly BRAF fusions, using current sequencing platforms. In contrast, BRAF V600E point mutations are more reliably detected using droplet digital PCR–based approaches [97,98].

Beyond diagnosis, CSF ctDNA shows promise for longitudinal monitoring. Emerging data demonstrate correlations between serial ctDNA measurements and radiographic disease course, treatment response, and early detection of recurrence. Also, newer strategies to analyze CSF are emerging, including assessment of methyloma, proteomics and metabolomics [[99], [100], [101]]. However, evidence specific to pLGG remains limited, and prospective validation is required before routine clinical implementation [94,102].

### Artificial intelligence

AI has transitioned from exploratory research to early stages of standardized implementation in pediatric neuro-oncology. In 2025, the AI–Response Assessment in Pediatric Neuro-Oncology (AI-RAPNO) subcommittee released the first international guidelines addressing responsible development and clinical integration of AI-based tools in this field [103]. These efforts address a central challenge in pLGG management: substantial inter- and intra-observer variability in manual tumor measurements, exacerbated by diffuse growth patterns, non-enhancing lesions, and complex anatomic locations.

Automated three-dimensional segmentation algorithms have demonstrated improved reliability and reproducibility compared with bidimensional Response Assessment in Neuro-Oncology (RANO) based measurements, with reported agreement rates approaching expert volumetric ground truth and clinically meaningful discordance in response classification identified in up to 20–30% of cases [[104], [105], [106]]. This precision is particularly relevant for distinguishing true progression from slow volumetric expansion typical of pilocytic astrocytomas [107].

Radiomic classifiers have also shown promise for non-invasive prediction of molecular subtypes, including differentiation between BRAF fusion–driven and BRAF V600E–mutant tumors, with reported performance metrics in the moderate-to-high range [108,109]. While these tools may support early risk stratification prior to molecular confirmation, external validation remains limited. Evidence for prediction of chemotherapy or targeted therapy response in pLGGs remains limited [108,109].

Future priorities include development of multimodal models integrating imaging, molecular, and clinical data, prospective validation in real-world settings, and implementation of explainable AI frameworks capable of generating clinically interpretable outputs. Key challenges include the need for large, harmonized pediatric datasets and standardized imaging protocols to support safe clinical deployment [103,104].

### Advanced imaging technologies

Beyond conventional anatomic MRI and AI-driven analysis, metabolic imaging and intraoperative technologies are increasingly explored to address diagnostic uncertainty and optimize surgical outcomes. The 2022 joint EANM/SIOPE/RAPNO guidelines has endorsed amino acid positron emission tomography (PET) imaging, particularly O-(2-[^18^F]fluoroethyl)-l-tyrosine (FET), as a problem-solving tool in pLGGs [110], FET-PET may aid in identifying biologically active regions within non-enhancing tumors, informing biopsy targeting and assisting in the differentiation of progression from treatment-related change [111]. However, uptake is variable in LGGs, and most pediatric data derive from mixed CNS tumor cohorts rather than pLGG-specific populations. While FET-PET parameters may assist in distinguishing progression from pseudo progression in the setting of cytostatic targeted therapies, evidence specific to pLGG remains limited [112].

In contrast, intraoperative MRI has demonstrated clearer benefit. Contemporary data confirm that routine use of intraoperative imaging significantly increases rates of gross total resection in pediatric LGGs without increasing neurological morbidity, supporting its role in the surgical management of resectable lesions [113,114]. Fluorescence-guided surgery using 5-aminolevulinic acid has limited utility in pLGG, with low rates of reliable fluorescence in WHO grade I–II tumors, and is not recommended for routine visualization [115,116].

### Digital health and remote monitoring

Visual impairment remains a major source of morbidity in optic pathway gliomas, highlighting the need for improved functional monitoring. Conventional visual field testing is often unreliable in young children due to developmental and attentional limitations. Virtual reality–based visual field testing has demonstrated higher completion rates and comparable diagnostic performance to standard perimetry in pediatric populations [117]. These systems employ gamified interfaces that may improve compliance, although further validation specifically in pLGG populations is required [118].

At present, validated tools for remote, home-based longitudinal monitoring of visual function in children with pLGG are lacking. Smartphone-based visual acuity applications demonstrate good correlation with standard testing in general pediatric cohorts but have not been validated for longitudinal use in optic pathway gliomas [119]. Current RAPNO guidelines continue to recommend standardized in-clinic visual assessments during active treatment [3]. Optical coherence tomography has emerged as a valuable objective biomarker correlating with visual outcomes in NF1-associated optic pathway gliomas, though it remains clinic-based. Development of validated remote monitoring tools tailored to this population remains an important unmet need [120].

### Immunologic and vaccine-based approaches

The immune microenvironment of pLGG is increasingly recognized as biologically relevant, though it remains incompletely characterized. Pilocytic astrocytomas are enriched for immune infiltrates, including tumor-associated macrophages and cytotoxic T cells, with MAPK-driven tumors demonstrating increased expression of TIM-3 and low expression of canonical immune checkpoint markers such as PD-1 and PD-L1 [[121], [122], [123]].

TIM-3 functions as a co-inhibitory receptor associated with T-cell exhaustion and immune suppression. Preclinical studies have demonstrated that TIM-3 blockade can reprogram the tumor immune microenvironment, enhancing antitumor immune activity and improving survival in murine models of BRAF fusion–driven glioma [122,124]. These findings have prompted early-phase clinical investigation of combined MAPK inhibition and immune checkpoint blockade, although clinical efficacy in pLGG remains to be established [121,124].

Vaccine-based strategies targeting glioma-associated antigens have also been explored. A pediatric pilot study demonstrated feasibility and encouraging progression-free survival in children with recurrent pLGG; however, data remain limited and such approaches are not currently practice-defining. Cellular therapies, including CAR T-cell approaches, remain investigational and are presently focused on high-grade gliomas [125,126].

## Ongoing challenges

Despite considerable advances, challenges remain in the management of pLGG. Access to molecular diagnostics and targeted therapies remains uneven globally, with resource limitations excluding many patients from precision-based care. Even within high-income settings, prolonged targeted therapy can impose significant financial toxicity on families [127,128].

Additionally, clinical uncertainty persists regarding optimal treatment duration and discontinuation strategies. MAPK inhibitors often require prolonged administration, and abrupt cessation can be associated with tumor regrowth, likely reflecting pathway reactivation in senescent tumor cells [4]. Evidence-based weaning strategies remain undefined [4,18].

Finally, the long-term sequelae of extended pathway inhibition in developing children are incompletely understood. While growth suppression associated with RAF and MEK inhibitors appears reversible in many patients, long-term cardiovascular and metabolic risks remain unknown, underscoring the need for lifelong survivorship surveillance. Integration of emerging technologies into routine practice will require careful validation, standardization, and health-system support to ensure equitable and safe implementation [129,130].

## Conclusion

This review shows that contemporary pLGG care has shifted from uniform treatment pathways toward individualized, longitudinal management of a biologically heterogeneous, largely MAPK-driven group of diseases. Molecular classification and the clinical integration of targeted therapies have redefined practice, prioritizing durable disease control and preservation of function across prolonged disease courses. However, key uncertainties remain, including optimal treatment duration, long-term toxicity, and strategies for tumors without actionable targets. Continued progress will depend on integrating germline predisposition into routine care, validating emerging diagnostic and monitoring tools, generating pediatric-specific evidence to guide sequencing and discontinuation, and improving equitable access to diagnostics and therapies.

## Author contributions

We confirm that this manuscript has not been previously published and is not under consideration elsewhere. All authors have contributed substantially to the work and have reviewed and approved the final manuscript.

## Declaration of competing interest

• Meziane Brizini – No conflict of interest
• Sébastien Perreault – Advisory boards, Alexion, Ipsen, Servier, Bayer. Grant support Bayer, and Roche
• Julie Bennett – Advisory boards for Alexion Canada (2025 and 2026), Servier Canada (2022 and 2024) and Rhythm Pharmaceuticals (2025)
• Hadeel Hassan – No conflict of interest
